# Whole blood lactate kinetics in patients undergoing quantitative resuscitation for septic shock

**DOI:** 10.1186/cc9690

**Published:** 2011-03-11

**Authors:** MA Puskarich, S Trzeciak, N Shaprio, A Heffner, JA Kline, AE Jones

**Affiliations:** 1 Carolinas Medical Center, Charlotte, NC, USA; 2 Cooper University Hospital, Camden, NJ, USA; 3 Beth Israel Deaconess Medical Center, Boston, MA, USA

## Introduction

We sought to compare the association of whole blood lactate kinetics with survival in patients with septic shock undergoing early quantitative resuscitation.

## Methods

Preplanned analysis of a multicenter emergency department (ED)-based randomized control trial of early sepsis resuscitation targeting three physiological variables: central venous pressure, mean arterial pressure, and either central venous oxygen saturation or lactate clearance. Inclusion criteria: suspected infection, two or more systemic inflammatory response syndrome criteria, and either SBP * <*90 mmHg after a fluid bolus or lactate >4 mmol/l. All patients had a lactate measured initially and subsequently at two hours. Normalization of lactate was defined as a lactate decline to <2.0 mmol/l in a patient with an initial lactate ≥2.0. Absolute lactate clearance (initial - delayed value), and relative ((absolute clearance)/(initial value) × 100) were calculated if the initial lactate was ≥2.0. The primary outcome was in-hospital survival. Receiver operating characteristic (ROC) curves were constructed and the area under the curve (AUC) was calculated. Differences in proportions of survival between the two groups at different lactate cutoffs were analyzed using 95% confidence intervals and Fisher exact tests.

## Results

Of 272 included patients, median initial lactate was 3.1 mmol/l (IQR 1.7, 5.8), and median absolute and relative lactate clearance were 1 mmol/l (IQR 0.3, 2.5) and 37% (IQR 14, 57). An initial lactate >2.0 mmol/l was seen in 187/272 (69%), and 68/187 (36%) patients normalized their lactate. Overall mortality was 19.7%. AUCs for initial lactate, relative lactate clearance, and absolute lactate clearance were 0.70, 0.69, and 0.58, respectively. Lactate normalization best predicted survival (OR = 6.1, 95% CI = 2.2 to 21), followed by lactate clearance of 50% (OR = 4.3, 95% CI = 1.8 to 10.3), initial lactate of <2 mmol/l (OR = 3.4, 95% CI = 1.5 to 7.8), and initial lactate <4 mmol/l (OR = 2.3, 95% CI = 1.3 to 4.3), with lactate clearance of 10% not reaching significance (OR = 2.3, 95% CI = 0.96 to 5.6).

## Conclusions

In ED sepsis patients undergoing early quantitative resuscitation, normalization of serum lactate during resuscitation was more strongly associated with survival than any absolute value or absolute/relative change in lactate. Further studies should address whether strategies targeting lactate normalization leads to improved outcomes.

